# Antioxidant and Antiproliferative Activity of *Allium ursinum* and Their Associated Microbiota During Simulated *in vitro* Digestion in the Presence of Food Matrix

**DOI:** 10.3389/fmicb.2020.601616

**Published:** 2020-12-01

**Authors:** Nemanja Stanisavljević, Svetlana Soković Bajić, Živko Jovanović, Ivana Matić, Maja Tolinački, Dušanka Popović, Nikola Popović, Amarela Terzić-Vidojević, Nataša Golić, Vladimir Beškoski, Jelena Samardžić

**Affiliations:** ^1^Laboratory for Molecular Microbiology, Institute of Molecular Genetics and Genetic Engineering, University of Belgrade, Belgrade, Serbia; ^2^Faculty of Biology, University of Belgrade, Belgrade, Serbia; ^3^Institute of Oncology and Radiology of Serbia, Belgrade, Serbia; ^4^Faculty of Chemistry, University of Belgrade, Belgrade, Serbia; ^5^Laboratory for Plant Molecular Biology, Institute of Molecular Genetics and Genetic Engineering, University of Belgrade, Belgrade, Serbia

**Keywords:** ramson, antiproliferative, antioxidant –phytochemical studies, probiotic, immunomodulation

## Abstract

In this study, for the first time, the comprehensive analysis of antiproliferative and antioxidant activities of ramson, followed by the analysis of its associated microbiota and health-promoting effects of lactic acid bacteria (LAB), was performed. Ramson (*Allium ursinum*) is recognized as a medicinal plant with a long history of use in traditional medicine due to its antimicrobial and antioxidant activity. In this study the influence of *in vitro* gastrointestinal digestion on the cytotoxic activity of *A. ursinum* extracts against human malignant cell lines was demonstrated. Seven sulfur compounds, the degradation products of thiosulfinates, including diallyl disulfide were shown to inhibit proliferation of malignant cells by inducing accumulation within G2/M phase as well as to induce apoptosis through activation of caspase-3 and mitochondrial signaling pathway. Further, the *A. ursinum* microbiota, particularly LAB with potential probiotic effects, was analyzed by culture-dependent method and culture-independent method [denaturing gradient gel electrophoresis (DGGE)]. The obtained results revealed that the most abundant genera were *Streptococcus*, *Lactobacillus*, and *Bacillus*. The *Lactobacillus* genus was mainly represented by *L. fermentum.* The pulsed-field gel electrophoresis (PFGE) analysis revealed the presence of two PFGE pulsotypes. The probiotic potential of the strain *L. fermentum* BGSR163 belonging to PFGE pulsotype 1 and the strain *L. fermentum* BGSR227 belonging to the PFGE pulsotype 2 was characterized. The results revealed that both strains are safe for human use, successfully survive the simulated gastrointestinal conditions, have potential to transiently colonize the gastrointestinal tract (GIT) and have a protective immunomodulatory effect, inducing the production of proinflammatory cytokine IL17 and regulatory cytokine IL10, while decreasing the production of proinflammatory cytokine IFN-γ. In conclusion, the results of this study suggest that consumption of *A. ursinum* might have health-promoting properties, including anticancer effects, while *L. fermentum* strains isolated from *A. ursinum* leaves could be used as probiotics for human consumption.

## Introduction

Ramsons or wild garlic (*Allium ursinum*) belongs to medicinal plant species which has been recognized as an important source of biologically active compounds, with the long history of application in traditional medicine as a natural remedy for gastrointestinal, cardiovascular, and respiratory disorders (Sobolewska et al., 2015). However, its chemical composition and biological activities have been rarely studied to date. The most important ramsons chemical constituents with pharmacological activity are undoubtedly sulfur compounds; among the most common are glutamyl peptides and sulfoxides, which are at the same time the most widely studied chemicals in this plant species. *Allium* species are characterized by high content of S-alk(en)yl-L-cysteine-sulfoxides which after hydrolysis yields many different volatile compounds including thiosulfinates and polysulfides responsible for characteristic odor and flavor (Sobolewska et al., 2015). The quantitative profile of cysteine-sulfoxides which could be found in ramson is highly variable depending on plant organ and harvest time. The most abundant sulfoxides are: methiin [(+)-S-methyl-L-cysteine-sulfoxide], aliin [(+)-S-2-propenyl-L-cysteine-sulfoxide], isoaliin [(+)-S-(1-propenyl)-L-cysteine-sulfoxide], propiin [(+)-S-propyl-L-cysteine-sulfoxide], as well as ethiin (S-ethyl-cysteine-sulfoxide). Tissue damage induced by processing or pathogen attack leads to hydrolytic cleavage of sulfoxides by C,S-lyases and formation of thiosulfinates responsible for the characteristic odor, as well as pyruvic acid and ammonia. The most abundant thiosulfinates present in ramson extracts are allicin (diallylthiosulfinate) and methyl-allyl- or dimethyl thiosulfinates, regarded as unstable and reactive which could be easily decomposed to dithiins, sulfides, polysulfides, ajoenes and many other volatile or non-volatile compounds even at room temperature. Although the composition, stability, as well as bioavailability of thiosulfinates from fresh garlic and several garlic products have been already examined (Lawson and Gardner, 2005; Rahman, 2007; Lawson and Hunsaker, 2018), this type of study has never been undertaken on *A. ursinum* fresh leaves. For that purpose, a standardized static *in vitro* digestion model described by Minekus et al. (2014) has been applied in this study to simulate the digestion of ramsons leaves in the presence of complex food matrix. Further, bioaccessible fraction was examined using two-dimensional gas chromatography to assess decomposition of thiosulfinates. In addition, antiproliferative and antioxidant activities were determined in the same fractions.

Despite the increased global interest in bioactive metabolites from medicinal plants, very little is known about the health-promoting effects of plant-associated microbiota. A range of bacterial species colonizes the rhizosphere, plant surfaces and internal tissues (epiphytes or endophytes) of various dietary and medicinal plants (Danhorn and Fuqua, 2007; Borozan and Raba, 2016; Presta et al., 2017). The microbiota composition from leaves, roots and rhizospheric soil was followed in aromatic plants *Thymus vulgaris* and *Thymus citriodorus* and correlated with the composition of essential oils (Checcucci et al., 2017). Further, the antimicrobial activities of bacteria isolated from root, leaf and fruit of medicinal plants *Echinacea purpurea* and *Peganum harmala* were tested (Presta et al., 2017; Bibi, 2017). The antimicrobial activities against various pathogenic bacteria were scored, such as *Escherichia coli*, methicillin-resistant *Staphylococcus aureus* (MRSA), *Enterococcus faecium*, *E. faecalis*, and *Pseudomonas aeruginosa*, indicating that medicinal plants could be a valuable source of bacteria with antimicrobial activity against clinically relevant pathogens (Bibi, 2017). In addition, the roles of endophytic bacterial strains of *Bacillus* sp., *Enterobacter* sp., *Pantoea* sp., *Erwinia* sp., and *Stenotrophomonas* sp. in the biosynthesis of various metabolites and therapeutic potential of *Fagonia indica* was reported (Rahman, 2007). Microbiota associated with *A. ursinum* has not been studied so far.

The composition of lactic acid bacteria (LAB) from *A. ursinum* fresh leaves was studied. Moreover, the range of lactobacilli species have been isolated, and their probiotic potential was characterized.

## Materials and Methods

### Isolation and Enumeration of LAB

The 10 g of fresh leaves was immersed in 90 ml of 0.85% saline and incubated for 3 h on 37°C with agitation. A volume of 1 ml of leaves and saline mixture was supplemented with 15% (w/v) glycerol, and later used for microbiological and microbiota analysis. After 3 h, 5 ml of mixture was added to 45 ml of 3 different media most suitable for isolation of LAB (MRS [Oxoid,], GM17 [Oxoid] and Nutrient broth[Oxoid]) and incubated for 72 h aerobically at 22°C and 30°C, and anaerobically (BD GasPak^TM^ EZ CO_2_ Container System, Becton, Dickinson and Company, Sparks, MD, United States) at 45°C. After incubation, serial dilutions (10^1^–10^7^) were prepared and plated onto MRS, GM17 and Nutrient agar with cycloheximide (1 μl/ml) for colony count. After the incubation approx. 30 colonies from each plate were selected randomly, streaked on new MRS, GM17, and Nutrient agar plates, respectively and examined under a microscope. The pure cultures were stored at −80°C in GM17, MRS or Nutrient broth supplemented with 15% (w/v) glycerol.

In parallel, 500 μl of each culture (grown in MRS, GM17 and Nutrient and from each temperature), was added to 10 ml of reconstituted skim milk (Merck GmbH, Darmstadt, Germany) and incubated for 48 h on three different temperatures as previously mentioned. After 48 h, the serial dilutions (10^1^–10^7^) of the isolates that curdled the skimmed milk were prepared. The rest of the experimental procedure was the same as the procedure mentioned above.

The 217 pure cultures were isolated and characterized according to their morphology by Gram staining and cell formation, catalase test and gelatinase production. Out of 217 isolates, 50 Gram-positive aerobically grown isolates were selected for further analysis. They were divided into 3 subgroups according to their growth on different mediums and different temperatures (23 isolates chosen from MRS plates incubated at 30°C, as well as 16 isolates chosen from GM17 plates and 11 isolates chosen from Nutrient-plates incubated at 22°C).

### DNA Isolation

Total DNA from pure cultures and first serial dilution of leaves and saline mixture were extracted as described by Hopwood et al. (1985).

### 16S rDNA Sequencing of LAB Isolates

16S rDNA PCR amplification was performed with UNI16SF (5′-GAGAGTTTGATCCTGGC-3′) and UNI16SR (5′-AGGAGGTGATCCAGCCG-3′) primers, complementary to 16S rRNA gene, as described by Jovcic et al. (2009). The obtained PCR amplicons were purified (Qiagen, GmbH, Hilden, Germany) and sequenced (Macrogen, Amsterdam, The Netherlands). To determine sequence similarities the BLAST algorithm was used in the NCBI nucleotide sequence database^1^.

### Denaturing Gradient Gel Electrophoresis Analysis and Fragment Sequencing

To investigate the dominant bacterial communities by denaturing gradient gel electrophoresis (DGGE) analysis, Quick-DNA Fecal/Soil Microbe Kit (Zymo Research, Irvine, CA, United States) was used for extraction of bacterial DNA from the first serial dilution of leaves and saline mixture. PCR amplification of the obtained DNA was performed using lactobacilli-specific primers Lab-0159f and Uni-0515-GCr (Invitrogen, Paisley, United Kingdom), as described by Lukic et al. (2013). The samples were amplified using KAPA *Taq* DNA polymerase (KAPA Biosystems, Cape Town, South Africa) in GeneAmp PCR System 2700 (Applied Biosystems, Foster City, CA, United States). DGGE analysis and gel manipulation after electrophoresis was performed according to Lukic et al. (2013). DGGE was performed on DGGE-2001 (C.B.S. Scientific, San Diego, CA, United States). Cloning of PCR fragments of interest, the transformation of the DH5α competent cells and analysis of selected transformants was performed as previously described Kulas et al. (2019). Sequence annotation and the database searches for sequence similarities were performed with the BLAST tool available online.^1^.

### Pulsed-Field Gel Electrophoresis

*Lactobacillus* sp. isolates were analyzed by pulsed-field gel electrophoresis (PFGE) in order to determine differences among the strains. PFGE was carried out as previously described (Tenover et al., 1995). Briefly, PFGE with *Not*I-digested genomic DNA was performed for 18 h at 300 V at 9°C using a 2015 Pulsafor unit apparatus (LKB Instruments, Bromma, Sweden). Grouping of isolates based on the PFGE results was done using SPSS software package, version 20 (IBM Inc., Chicago, IL, United States).

### *In vitro* Digestion

Fresh leaves of *A. ursinum* were quickly homogenized in liquid nitrogen using mortar and pestle. (Oxoid, Hampshire, United Kingdom) medium, were harvested by centrifugation (4500 × *g*, 10 min) and washed in 0.85% saline. Aliquots of *A. ursinum* powder (2 g) or bacterial pellets, obtained from 10 ml of overnight *L. fermentum* BGSR163 and *L. fermentum* BGSR227 cultures in MRS were placed in 50 ml conical tubes and mixed with 3 g of infant formula (Juvitana, Swisslion Product d.o.o. Indjija, Serbia) which contained 3% of protein, 10% of carbohydrate and 1% of fat according to manufacturer’s specifications. The formula was prepared from boiled turkey meat, boiled potato and corn paste, rice flour, and water. Control was prepared by mixing 2 ml of distilled water with 3 g of infant formula. Obtained mixtures were subjected to standardized static *in vitro* digestion according to the method of Minekus et al. (2014). Oral phase of simulated digestion was started by addition of 5 g of previously obtained mixture to 3.5 ml of simulated salivary fluid (SSF), 0.5 ml of salivary α-amylase solution (1500 U/ml), 25 μl of 0.3 M CaCl_2_ and 975 μl of water, shaken vigorously and incubated 2 min at 37°C. In the following gastric phase, 10 ml of previously treated oral bolus was mixed with 7.5 ml of simulated gastric fluid (SGF), 1.6 ml of pepsin solution (25000 U/ml), prepared in SGF, 5 μl of 0.3 M CaCl_2_ and pH was adjusted to 3.0 using 1M HCl. The mixture was supplemented with distilled water to achieve volume of 20 ml. Obtained mixture was incubated at 37°C for 2 h, using orbital shaker at 300 rpm. For the final intestinal phase 20 mL of obtained gastric chyme was mixed with 11 ml of simulated intestinal fluid (SIF), 5 ml of pancreatin solution 800 U/ml (trypsin activity) prepared in SIF, 2,5 ml of 160 mM bile salt solution and 40 μl of 0.3 M CaCl_2_. The pH value of 7.0 was adjusted using 1M NaOH and the mixture was supplemented with distilled water to reach final volume of 40 ml. Additional incubation was performed with constant shaking at 300 rpm during 2h at 37°C. After completed digestion samples were centrifuged at 4500 × *g* for 10 min at 4°C, supernatants were kept and their volume was accurately measured, then immediately frozen in liquid nitrogen and kept for further experiments at −80°C. Composition of SSF, SGF and SIF electrolyte solutions was presented in Table 1. The results of *L. fermentum* BGSR163 and *L. fermentum* BGSR227 survival after *in vitro* digestion were expressed as Log CFU (Colony forming units)/ml and the survival rate was calculated from the viable cell count with respect to initial cell counts. The aliquots were taken at 0, 10, 120, and 220 min of the simulated digestion. The 10 times serial dilutions were prepared in 0.85% saline and plated on MRS agar plates and incubated anaerobically at 37°C for 48 h. The experiments were performed in triplicates.

**TABLE 1:** Preparation of stock solutions and simulated digestive fluids.

Compound	Stock conc.		SSF	SGF	SIF
			pH 7.0	pH 3.0	pH 7.0
			Conc. in SSF	Conc. in SGF	Conc. in SSF
	**g/l**	**mol/l**	**mmol/l**	**mmol/l**	**mmol/l**
KCl	37.3	0.5	15.1	6.9	6.8
KH_2_PO_4_	68	0.5	3.7	0.9	0.8
NaHCO_3_	84	1	13.6	25	85
NaCl	117	2	–	47.2	38.4
MgCl_2_(H_2_0)_6_	30.5	0.15	0.15	0.1	0.33
(NH_4_)_2_CO_3_	48	0.5	0.06	0.5	–
NaOH	–	1	–	–	8.4
HCl	–	6	1.1	15.6	–
CaCl_2_ (H_2_O)_2_	44.1	0.3	(0.75)	(0.075)	(0.3)

*SSF, simulated salivary fluid; SGF, simulated gastric fluid; SIF, simulated intestinal fluid.*

### Comprehensive Two-Dimensional Gas Chromatography/Mass Spectrometry (GC × GC-MS)

For GC × GC-MS analysis, obtained samples after *in vitro* digestion of *A. ursinum* were extracted using dichloromethane (CH_2_Cl_2_) for 1 h by liquid/liquid extraction. Prepared extracts were filtered using membrane filter (0.45 μm, Agilent) and after concentration directly analyzed by GC × GC-MS GCMS-QP2010 Ultra (Shimadzu, Kyoto, Japan) equipped with ZX2 thermal modulation system (Zoex Corp.) as Total Ion Chromatograms (TIC). A Rtx^®^-1 (first column: RESTEK, Crossbond^®^ 100% dimethyl polysiloxane, 30 m, 0.25 mm ID, df = 0.25 μm) and a BPX50 (SGE Analytical Science, 1 m, 0.1 mm ID, df = 0.1 μm) columns were connected using the GC × GC modulator as the first and second capillary columns, respectively. The oven was programmed at an initial temperature of 40°C for 4 min, then ramped at 10°C/min to 310°C. Isothermal step at 310°C for 5 min was at the program end. The modulation period was 6 s with 300 ms of hot jet pulse. The MS data were collected using Shimadzu GC/MS Real-Time Analysis and GC × GC-MS data were analyzed using GC Image R2.6 (GC Image LLC). Obtained MS spectra were compared with the MS libraries NIST 11, NIST 11s, and Wiley 8.

### Total Phenolic Content (TPC)

Total phenolic content was determined according to the previously described method of Julkunen-Tiitto (1985) with slight modifications. Aliquots of supernatants obtained after *in vitro* digestion (20 μl) were mixed with 1580 μl of distilled water and 100 μl of Folin–Ciocâlteu reagent and shaken vigorously. After 15 min incubation at room temperature, 300 μl of 20% sodium carbonate solution was added to the mixture which was then incubated for additional 2 h. After the incubation absorbance of the mixture was recorded at 765 nm. Total phenolic content was calculated using gallic acid standard curve and expressed as mg of gallic acid equivalents per ml of supernatant (mg GAE/ml).

### Total Flavonoid Content (TFC)

Supernatants collected after *in vitro* digestions were concentrated five times by evaporating under vacuum at 60°C. Obtained concentrates were then centrifuged at 5000 × *g* for 15 min to precipitate insoluble particles. The total flavonoid content in the concentrated supernatants was then determined by aluminum chloride assay according to the previously described method of Ribeiro et al. (2015) with modifications. Supernatants (125 μl) were mixed with 625 μl of distilled water and 37.5 μl 5% NaNO_2_. After 6 min of incubation at room temperature, 75 μl of 10% AlCl_3_ solution was added and incubation was prolonged for 5 min. After the second incubation, 250 μl of 1M NaOH was added and mixture was supplemented with distilled water to a final volume of 1.25 ml. Absorbance of the samples was recorded at 510 nm. The flavonoid content was calculated using quercetin standard curve (5–100 μg/ml) and expressed as μg of quercetin equivalents per mL of supernatant (μg QE/ml).

### Measurement of Relative DPPH Scavenging Capacity (RDSC)

Relative 2,2-Diphenyl-1-Picrylhydrazyl (DPPH) radical scavenging capacity measurement was determined according to Cheng et al. (2006). The reaction mixture consisted of 100 μL of supernatant diluted with methanol. Five different concentrations (10, 20, 40, 60, and 80 μL) of supernatant and Trolox were used. Then, 100 μL of 200 μM DPPH in methanol was added to each microplate and shaken. Blank contained 200 μL of methanol and control sample was prepared by mixing 100 μL of methanol and 100 μL of DPPH solution. Absorbance was continuously recorded each minute for 1.5 h at 515 nm. The percent of DPPH quenched for each time point was calculated according to the equation:

$$\%\mathrm{DPPH}\mathrm{quenched}=[1-\left(A_{\mathrm{sample}}-A_{\mathrm{blank}}\right)/\left(A_{\mathrm{control}}-A_{\mathrm{blank}}\right)]\times 100$$

The values of DPPH in percents quenched in different time points for each extract and standard were plotted against reaction time. The area under obtained curve (AUC) was determined according to the following equation:

$$\text{AUC}=0,5\,f_{0}+\left(f_{1}+f_{2}+f_{3}+\ldots +f_{i-1}\right)+0,5\,f_{i}$$

Where *f*_0_ represents DPPH quenched at the start of the measurement and *f*_*i*_ is DPPH quenched at reaction time *i*. Relative DPPH radical scavenging capacity (RDSC) was calculated according to the equation:

$$\mathrm{RDSC}\,\left(\mathrm{mM}\,\mathrm{TE}/\mathrm{ml}\right)={\mathrm{AUC}}_{\mathrm{sample}}/{\mathrm{AUC}}_{\mathrm{trolox}}\times {\mathrm{molarity}}_{\mathrm{trolox}}/{\mathrm{volume}}_{\mathrm{sample}}$$

### Ferrous Ion Binding Capacity

The ferrous ion binding capacity was monitored by the previously described method of Dinis et al. (1994) with slight modifications. Briefly, 940 μL of supernatant dilutions in distilled water were mixed with 20 μL of 2 mM ferrous chloride and incubated for 30 min at room temperature. After incubation, 200 μL of 5 mM ferrozine was added and the mixture was incubated for 10 min under the same conditions. Formation of a red ferrous ion-ferrozine complex was recorded at 562 nm. Control sample was prepared with distilled water instead of the sample. Percentage of ferrous ion binding was calculated according to the equation:

$$\%\mathrm{of}\mathrm{bound}{\mathrm{Fe}}^{2}+=\left(A_{\mathrm{control}}-A_{\mathrm{sample}}\right)/A_{\mathrm{control}}\times 100$$

### *In vitro* Cytotoxic Activity

The cytotoxic effects of the undigested and digested *A. ursinum* extracts were investigated on human cervical adenocarcinoma HeLa and human colon adenocarcinoma LS174 cell lines (American Type Culture Collection, Manassas, VA, United States). The cells were cultured in the complete nutrient medium (RPMI 1640 supplemented with L-glutamine (2 mM), streptomycin (100 μg/ml) and penicillin (100 IU/ml), 10% heat-inactivated fetal bovine serum (FBS) and 25 mM HEPES), as described elsewhere (Matić et al., 2013). HeLa (2,000 cells per well) and LS174 (7,000 cells per well) cells were seeded in 96-well microtiter plates. The cells were allowed to adhere for 24 h, and afterward, five concentrations of tested extracts were added to the cells ranging from 1.25% to 20% (five serial two-fold dilutions). The nutrient medium was added to the control cells. Cell survival was determined by MTT assay after 72 h treatment, according to the procedure firstly described by Mosmann (1983) and modified by Ohno and Abe (1991). All experiments were done in triplicate. Cisplatin was used as a positive control. The IC_50_ concentration was defined as the concentration of an extract causing the inhibition of cell survival by 50% compared with a control cell sample.

### Cell Cycle Analysis

HeLa and LS174 cells were exposed to extracts of *A. ursinum* for 24 h (applied concentrations were 5% for HeLa and 20% for LS174 cells). After incubation the cells were collected, washed in phosphate buffered saline (PBS) and fixed in 70% ethanol on ice, according to a standard protocol (Ohno and Abe, 1991; Ormerod, 1994). Cell samples were stored at −20°C for at least one week before staining. The cells were incubated with RNase A in PBS (final concentration 200 μg/ml) for 30 min at 37°C. Next, the propidium iodide, at a concentration of 40 μg/ml, was added to the cells. Cell cycle phase distributions were assessed by FACSCalibur Flow Cytometer (BD Biosciences, Franklin Lakes, NJ, United States). CELLQuest software (BD Biosciences) was used for data analyses.

### Effects of Specific Caspase-3 Inhibitor

To examine whether *A. ursinum* extracts could activate apoptosis in HeLa cells, the specific irreversible peptide inhibitor of caspase-3 (Z-DEVD-FMK, R&D Systems, Minneapolis, United States) was used for cell cycle analysis, as described previously (Matić et al., 2013). Briefly, HeLa cells were preincubated for 2 h with Z-DEVD-FMK at a final concentration of 40 μM. Afterwards, the cells were treated with extracts (applied at a concentration of 5%). After 24 h treatment, the cells were harvested and fixed in 70% ethanol. Cell cycle analysis was performed as described in the previous section.

### Gelatinase Activity

Gelatinase activity was assayed according to the procedure described by Su et al. (1991). The bacterial isolates were plated on agar plates containing 3% gelatin (Oxoid) at 37°C for 48 h and flooded with a saturated solution of ammonium sulfate (Centrohem, Stara Pazova, Serbia). A transparent halo around cells and gelatin precipitates was used as an indication for gelatinase producers. *E. faecalis* V583 (Paulsen, 2003) was used as a positive control.

### Hemolytic Activity

The two *L. fermentum* (BGSR163 and BGSR227) isolates were tested for hemolytic activity. The strains were cultured in MRS broth and were streaked on Columbia agar plates containing 5% of sheep blood (Oxoid). The plates were incubated for 48 h at 37°C. Blood agar plates were examined for signs of β-hemolysis (clear zones around colonies), α-hemolysis (green-hued zones around colonies) or γ-hemolysis (no zones around colonies) as described Argyri et al. (2013).

### Minimal Inhibitory Concentration (MIC)

Minimal inhibitory concentrations (MICs) of clinically relevant antibiotics were determined by microdilution testing following European Food Safety Authority (EFSA, Panel, 2012) guidance. Susceptibility was tested against: ampicillin (2 μg/ml), gentamicin (16 μg/ml), kanamycin (64 μg/ml), streptomycin (64 μg/ml), erythromycin (1 μg/ml), clindamycin (1 μg/ml), tetracycline (8 μg/ml), and chloramphenicol (4 μg/ml). Microdilution tests were performed in MRS (Oxoid). Cell density was monitored after 24 h incubation at 37°C at 595 nm using Plate Reader Infinite 200 pro (MTX Lab Systems, Vienna, Austria).

### Cytotoxicity Assay of Probiotic Strains and Theirs Postbiotics

Lactate dehydrogenase (LDH) Cytotoxicity Assay Kit (Thermo Fisher Scientific, Waltham, MA, United States) was used for a measure of cytotoxicity level in the cell cultures through detection of LDH released from dead cells. After treatments of Caco-2 and mesenteric lymph node cells (MLNC) with selected *Lactobacillus* strains, LDH activity in supernatants was determined by following the manufacturer’s instructions. The absorbance was measured at 450 nm using Plate Reader Infinite 200 pro (MTX Lab Systems).

### Adherence to Caco-2 Cells

The adhesion ability of *L. fermentum* isolates was performed on colonocyte-like Caco-2 cells. Caco-2 was purchased from the European Collection of Cell Cultures (ECACC No. 86010202). The culture and maintenance of the cells were done according to procedures described by Sánchez et al. (2010) using Dulbecco’s Modified Eagle Medium (DMEM) supplemented with 10% fetal bovine serum (FBS), 100 U/ml penicillin and 100 mg/ml streptomycin and 2 mM l-glutamine. Media and reagents were purchased from Thermo Fisher Scientific. The adherence (expressed as a percentage) was calculated as: CFU adhered bacteria/CFU added bacteria. Experiments were performed in two replicated plates and in each plate; three wells were used per sample.

### Isolation and Treatment of Mesenteric Lymph Node Cells

Mesenteric lymph nodes (MLN) were isolated from Wistar rats and the suspension of cells (MLNC) was prepared by mechanical disruption of MLN, as described at Bajić et al. (2020). MLNC were stimulated with concanavalin A (ConA, Sigma-Aldrich, 2.5 μg/ml) and treated with cell culture supernatants originating from 2.5 × 10^7^ bacterial cells, with UV inactivated bacteria at a ratio of 10:1 (bacteria to eukaryotic cell), 1% of *A. ursinum* extract after passing through the gastrointestinal tract (GIT), and complex food matrix used as a carrier while passing through the GIT for 72 h. After 72 h of incubation, the cell culture supernatants were collected and subjected to cytotoxicity assay and cytokines quantification.

All experimental procedures were approved by the Ethics committee of the Ministry of agriculture, forestry and water management of the Republic of Serbia (license number 119-01-5/14/2017-09).

### Cytokines Quantification

Cytokines concentration in 72 h cell culture supernatants was determined by enzyme-linked immunosorbent assay (ELISA). For interleukin (IL)-10 and interferon (IFN)-γ detection, specific DuoSet ELISA kits were used according to the manufacturer’s instructions (R&D Systems, Minneapolis, MN, United States). For IL-17A detection, rat IL-17A (homodimer) ELISA Ready-SET-Go was used according to the manufacturer’s instructions (eBioscience, San Diego, CA, United States). Samples were analyzed in duplicates and the results were calculated using the standard curves.

### Statistical Analysis

All data are presented as mean values ± standard error of the mean. One-way ANOVA with the Dunnett’s *post hoc* test was used to compare multiple groups. Values at *p* < 0.05 or less were considered statistically significant. All experiments were repeated at least three times. Statistical analysis and graph design were carried out using GraphPad Prism Software.

## Results

### Total Phenolic, Total Flavonoid Content and Antioxidant Activities

Total phenolic content (TPC) of the supernatants obtained before and after digestion were presented in Table 2. Bioaccessible fractions of undigested ramson SN1t_0_-SN3t_0_ contained 187–221 μg GAE/mL and food matrix alone had lower phenolic content of 92 μg GAE/ml, while digested samples showed significantly higher TPC values of 235–264 μg GAE/ml for ramson samples and 110 μg GAE/ml for food matrix. In case of all studied samples *in vitro* digestion provoked a statistically significant increase in TPC. Total flavonoid content of bioaccessible fraction before the digestion ranged from 35.1 to 46.2 μg QE/ml, while after the digestion, a slight increment was recorded in two ramsons samples SN1 and SN2. Bioaccessible fraction of undigested food matrix contained a significant content of flavonoids 15.8 μg QE/ml which was also increased after the digestion as it can be seen from Table 2. Antioxidant activity of undigested samples, measured by kinetic DPPH scavenging assay, followed the same trend as TFC, ranging from 0.35 to 0.45 μM TE/ml. The digestion provoked a significant elevation of radical scavenging in SN1 and SN2 samples. Food matrix showed twice lower activity for undigested sample and the increment after digestion was not observed. Ferrous ion chelating capacities before digestion ranged from 42.5%, detected in food matrix, up to 71.3% in SN2 sample. Substantial increase of FCC after digestion was recorded in all samples including food matrix.

**TABLE 2:** Total phenolic, flavonoid content, and antioxidant activities before and after *in vitro* digestion.

	TPC (μg GAE/ml)	TFC (μg QE/ml)	DPPH scavenging (μM TE/ml)	FCC (%)
SN1 t_0_	221 ± 2	46.2 ± 1.6	0.45 ± 0.05	68.2 ± 0.9
SN1 – after digestion	264 ± 1*	55.4 ± 1.1*	0.59 ± 0.07*	85.0 ± 1.6*
SN2 t_0_	205 ± 3	35.1 ± 0.8	0.40 ± 0.06	71.3 ± 1.9
SN2 – after digestion	235 ± 2*	43.3 ± 0.5*	0.56 ± 0.05*	88.1 ± 0.8*
SN3 t_0_	187 ± 4	38.0 ± 1.6	0.35 ± 0.07	66.5 ± 1.4
SN3 – after digestion	239 ± 2*	40.4 ± 1.4	0.40 ± 0.06	86.6 ± 1.6*
SNM t_0_	92 ± 2	15.8 ± 0.9	0.19 ± 0.05	42.5 ± 2.1
SNM – after digestion	110 ± 1*	22.7 ± 1.1*	0.24 ± 0.05	76.8 ± 1.8*

SN, supernatants of *Allium ursinum* extracts obtained before and after digestion; SNM, solely food matrix; TPC, Total phenolic content; TFC, Total flavonoid content; DPPH, 2,2-Diphenyl-1-Picrylhydrazyl; FCC, ferrous ion chelating capacity; GAE, gallic acid equivalent; QE, quercetin equivalent; TE, trolox equivalent. Values are shown as means ± SD (*n* = 3); statistical significance *p < 0.05 (Student’s *t*-test).

### GC × GC-MS Analysis

**FIGURE 1:**
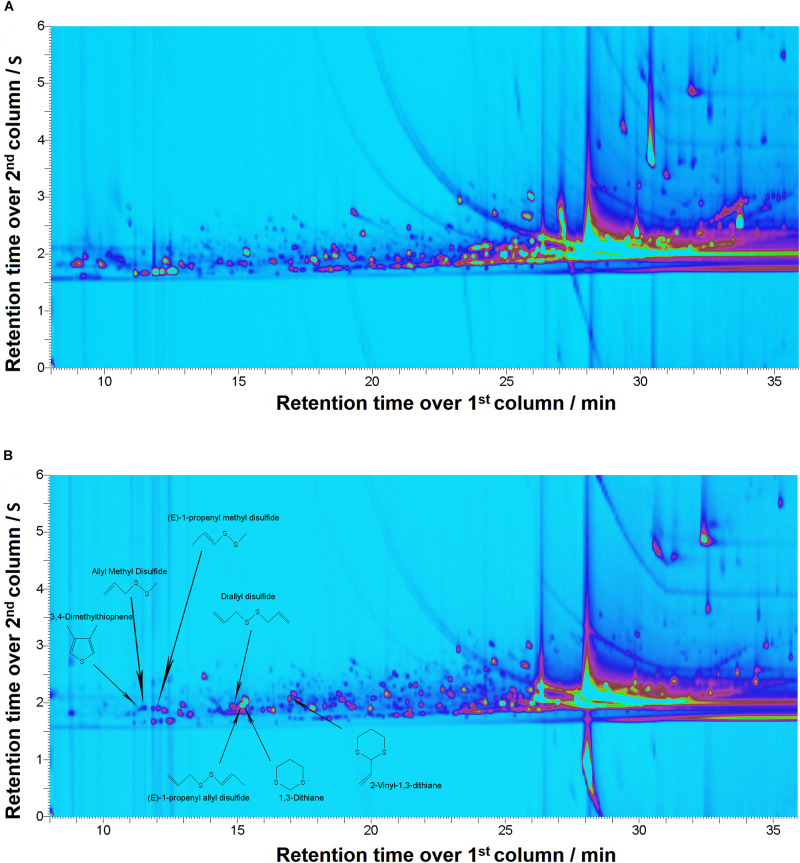
Comprehensive two-dimensional gas chromatography/mass spectrometry (GC × GC-MS). Figures depicting chromatograms for: **(A)** Supernatant of the digested food matrix; **(B)**. Supernatant of the digested sample SN1

Two-dimensional gas chromatography analysis of *A. ursinum* bioaccessible fraction obtained after digestion revealed presence of seven volatile compounds which were absent in supernatant of digested food matrix (Figures 1A,B). The dominant compounds in the analyzed spectrum of samples containing *A. ursinum* were allicin decomposition products: 3,4-dimethylthiophene; 1,3-dithiane; diallyl disulfide; allyl methyl disulfide; 2-vinyl-1,3-dithiane; (E)-1-propenyl methyl disulfide; (E)-1-propenyl allyl disulfide (Figure 1B).

### The Effects of the Extracts on Cell Cycle Phase Distribution

**FIGURE 2:**
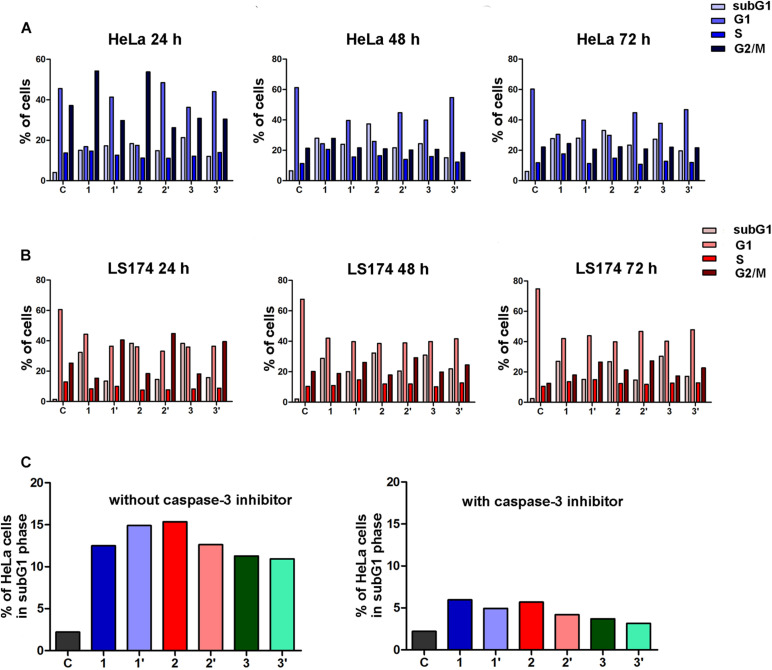
Changes in the cell cycle phase distribution of human cervical adenocarcinoma (HeLa) cells induced by the *Allium ursinum* extracts (*c* = 5%) after 24 h, 48 h, and 72 h of treatment **(A)**. Changes in the cell cycle phase distribution of human colon adenocarcinoma (LS174) cells induced by the *Allium ursinum* extracts (*c* = 20%) after 24 h, 48 h, and 72 h of treatment. **(B)** Effects of specific caspase-3 inhibitor on the percentages of subG1 HeLa cells exposed to *Allium ursinum* extracts for 24 h without caspase-3 inhibitor and pretreated with caspase-3 inhibitor before treatment with extracts **(C)** C-control HeLa or LS174 cells, extract 1 control for digestion, extract 1′ – digested, extract 2 control for digestion, extract 2′ – digested, extract 3 control for digestion, extract 3′ – digested.

Investigation of the mechanisms of the cytotoxic activity of the *A. ursinum* extracts by cell cycle analysis revealed that all tested extracts applied at a concentration of 5% induced remarkable increase of HeLa cells in the subG1 phase in comparison with those determined in control, untreated cell sample after treatments that lasted 24, 48, and 72 h. The effects of the extracts on cell cycle phase distribution are presented in Figure 2A. The differences between the percentages of HeLa cells at specific phases of the cell cycle exposed to undigested and digested *A. ursinum* extracts were observed. Undigested extracts **1** and **2** after 24 h incubation caused accumulation of HeLa cells within the G2/M cell cycle phase in comparison with control cells. The increase of cells in the S phase was observed in HeLa cells treated for 48 and 72 h with undigested extracts. Among undigested extracts, the extract 1 induced the most intensive accumulation of cells within the S phase, which was accompanied with G2/M phase arrest after 48 h treatment. The treatment with food matrix of HeLa cells did not significantly affect their cell cycle phase distribution (Figure 2A).

In human colon adenocarcinoma LS174 cells exposure for 24, 48 and, 72 h to undigested and digested *A. ursinum* extracts applied at a concentration of 20% led to remarkable increase in the percentage of subG1 cells when compared with control cell sample, as shown in Figure 2B. The increase of LS174 cells within the subG1 phase was remarkably higher in cells incubated with 20% undigested extracts. In addition, all three digested extracts caused G2/M cell cycle arrest in LS174 cells after 24, 48 and, 72 h of incubation. Accumulation of LS174 cells within the G2/M phase was the most prominent in cell samples exposed for 24 h to tested extracts. It should be noted that the food matrix at a concentration of 20% slightly increased the percentage of LS174 cells within the subG1 phase.

Cytotoxic activity of *A. ursinum* tested extracts digested *in vitro* on HeLa and LS174 cells is presented in Table 3.

**TABLE 3:** Cytotoxic activity of *Allium ursinum* extracts digested *in vitro.*

	HeLa	LS174
	
	*IC_50_ (mean* ± *standard deviation)*	
SN1 t_0_	2.75 ± 0.20	20
SN1	4.02 ± 0.13	> 20
SN2 t_0_	3.18 ± 0.26	20
SN2	4.38 ± 0.21	> 20
SN3 t_0_	4.15 ± 0.02	19.69 ± 0.44
SN3	4.90 ± 0.09	> 20
cisplatin	2.46 ± 0.08	13.99 ± 2.00

*IC_50_ values represent mean ± standard deviation of three independent experiments. Values for extracts are shown in percentages, values for cisplatin are shown in μM. SNt_0_ – supernatants of *A. ursinum* extracts obtained before digestion, SN – after digestion; HeLa-human cervical adenocarcinoma cell lines; LS174-human colon adenocarcinoma cell lines.*

### Effects of the Caspase-3 Inhibitor

To explore whether the observed increase of HeLa cells within the subG1 phase after 24 h exposure to undigested and digested *A. ursinum* extracts could be attributed to apoptotic cell death, the cell cycle analysis of cells pretreated with a specific inhibitor of caspase-3 was performed. The decrease in the percentage of subG1 HeLa cells pretreated with caspase-3 inhibitor before exposure to each of the tested extracts was found when compared with treated cells which were not preincubated with an inhibitor (Figure 2C). These results point to the ability of both undigested and digested extracts of *A. ursinum* to induce apoptosis in HeLa cells through activation of main effector caspase-3.

### Evaluation of *A. ursinum-*Associated Microbiota

**FIGURE 3:**
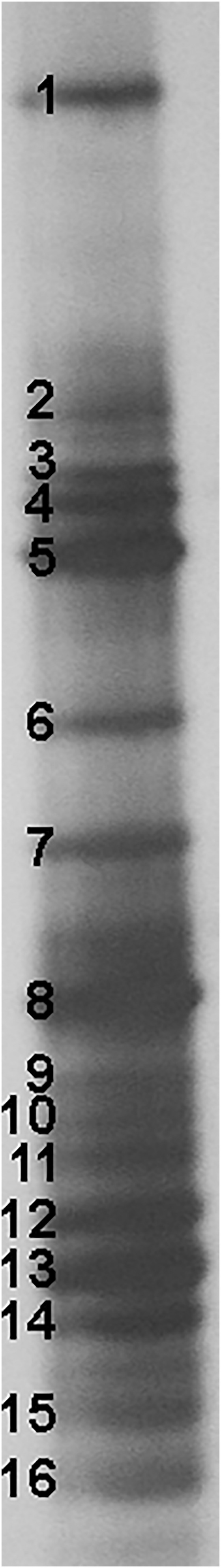
Denaturing gradient gel electrophoresis (DGGE) profiles of rDNA amplicons obtained using a *Lactobacillus*-specific primer set on bacterial DNA isolated from *Allium ursinum* leaf tissue samples. Bands indicated by numbers (1–16) were excised, cloned, and sequenced.

In order to determine the composition of LAB and closely related bacteria associated with *A. ursinum*, DGGE analysis of 16S rRNA amplicons obtained by using lactobacilli-specific primers Lab-0159f and Uni-0515-GCr was performed. DGGE analysis of the dominant microbiota present in the first dilution of *A. ursinum* leaves revealed the predominant presence of genera: *Streptococcus*, corresponded to the bands 5, 6, 7, 8, and 9 (69.2%), *Lactococcus*, corresponded to the bands 2, 3, and 4 (23.1%), and *Lactobacillus*, corresponded to the bands 11 and 13 (15.3%). Sequencing results of the re-amplified bands from DGGE gel indicated the domination of *Lactococcus lactis*, *Streptococcus thermophilus*, *Lactobacillus reuteri*, and *Lactobacillus gasseri* (Figure 3 and Supplementary Table 1).

### Isolation and Identification of LAB From *A. ursinum*

LAB and other closely related bacteria were isolated from the first dilution of *A. ursinum* leaves. The 50 isolates selected on the basis of their growth on MRS, GM17, and Nutri agar were further analyzed by 16S rDNA sequencing. The most abundant genus belonged to *Staphylococcus* (34%), *Lactobacillus* all of them *L. fermentum* (30%), and *Bacillus* (14%) (Supplementary Table 2).

**FIGURE 4:**
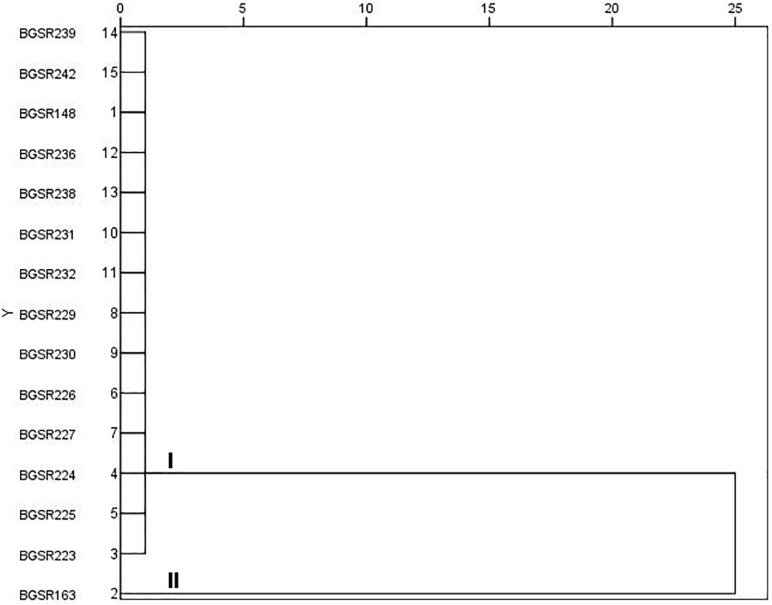
Dendrogram derived from *Not*I PFGE patterns showing the relatedness of *Lactobacillus fermentum* species isolated from *Allium ursinum*. The dendrogram was constructed using SPSS software. Letters I and II indicate two major clusters.

In order to determine inter-strain differences among the *L. fermentum* isolates, 15 *L. fermentum* strains were analyzed by PFGE. The dendrogram produced by SPSS software demonstrated genomic similarity ranging from 75 to 100% (Figure 4). Two clusters with differences up to 25% were observed. Cluster I comprise one pulsotype (14 isolates); while cluster II contains only one pulsotype with one isolate BGSR163. The band pattern revealed the presence of two different *L. fermentum* PFGE puslotypes (strains) (Figure 4), hence the strains BGSR163 and BGSR227, belonging to different PFGE pulsotypes, were chosen for cytotoxicity assay, as well as for evaluation of probiotic potential –survival through simulated GIT, adhesion to intestinal epithelial cells (IEC), and immunomodulatory activity, as the main criteria for selection of probiotic strains.

### Determination of the Safety Status of the *L. fermentum* BGSR163 and *L. fermentum* BGSR227 Isolates

**FIGURE 5:**
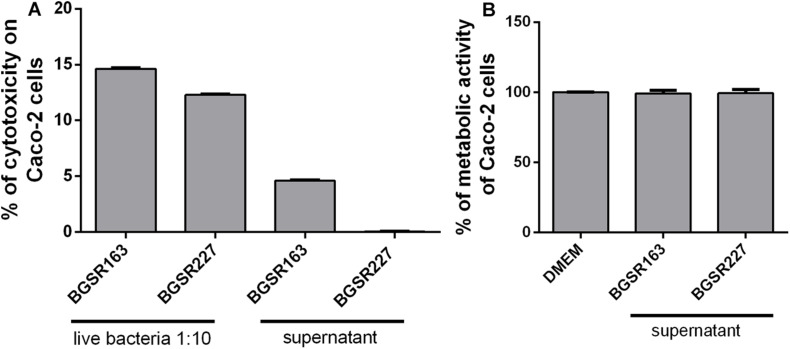
The effect of *Lactobacillus fermentum* BGSR163 and BGSR227 live strains and cell culture supernatants on toxicity, assessed by LDH assay **(A)** and the effect of *L. fermentum* BGSR163 and BGSR227 cell culture supernatant on metabolic activity of Caco-2 cells **(B)**. All values are presented as mean ± SD from three independent experiments. DMEM – Dulbecco’s Modified Eagle’s Medium.

In order to determine the safety status of the *L. fermentum* isolates, the cytotoxic, gelatinase, and hemolytic activities were assayed, and the susceptibility to minimal inhibitory concentrations of antibiotics was determined. The Caco-2 cells were exposed to live *L. fermentum* isolates to determine the possible cytotoxic effect of the strains. The results showed that *L. fermentum* BGSR163 and *L. fermentum* BGSR227 live bacterial strains exhibited cytotoxic activity to Caco-2 cells, while the cell culture supernatants from both strains did not exhibit cytotoxic activity on the same cells (Figure 5A). MTT assay showed that cell culture supernatants from *L. fermentum* BGSR163 and *L. fermentum* BGSR227 do not decrease cell metabolic activity of Caco-2 cells after the treatment (Figure 5B). Absence of gelatinase activity, for both *L. fermentum* strains (BGSR162 and BGSR227), on gelatin agar plates was noted, as well as absence of hemolysis zones, on blood agar plates. Both strains were susceptible to all clinically relevant antibiotics (ampicillin, gentamicin, kanamycin, streptomycin, erythromycin, clindamycin, tetracycline, and chloramphenicol).

### Survival of *L. fermentum* BGSR163 and *L. fermentum* BGSR227 in Chemically Simulated GIT Transit

**FIGURE 6:**
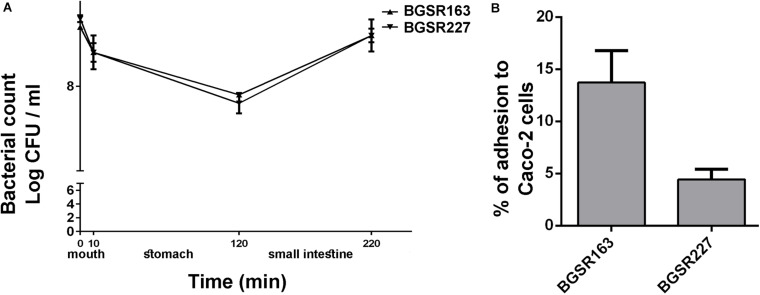
Survival of selected *Lactobacillus fermentum* BGSR163 and BGSR227 isolates, as indicated on the right, under oral conditions (0–10 min) gastric conditions (10–120 min), and intestinal conditions (120–220 min). All values are presented as mean ± SD from three independent experiments **(A)**. Adhesion of *Lactobacillus fermentum* BGSR163 and BGSR227 on Caco-2 cells. All values are presented as mean ± SD from three independent experiments. **(B)**.

The survival of the strains *L. fermentum* BGSR163 and *L. fermentum* BGSR227 in chemically simulated GIT transit was tested. The results revealed that both strains successfully survived the passage through simulated GIT conditions (Figure 6A). The results showed that the number of viable cells of both *L. fermentum* strains after 10 min (simulated mouth conditions) was not significantly changed compared to that at the beginning of the experiment. However, 110 min exposure to simulated gastric conditions lowered the number of viable cells of both *L. fermentum* strains for approx.1 log unit (ΔLogBGSR163 = 0.8 and BGSR227 = 1), although the number of viable cells was rather high (8 Log CFU/ml). Interestingly, when the cells were exposed to the simulated small intestine conditions (100 min) with increased bile salts concentrations, the growth of the strains was restored (ΔLogBGSR163 = 0.7 and ΔLogBGSR227 = 0.8). The survival rate of the strain BGSR163 was 98.85%; Δlog CFU/ml = 0.1), while the survival rate of BGSR227 was 97.73%; Δlog CFU/ml = 0.2 (Figure 6A).

### Adherence to Caco-2 Cells

The adherence abilities of the *L. fermentum* isolates BGSR163 and *L. fermentum* BGSR227 were investigated on Caco-2 cells. Both strains were able to adhere to Caco-2 cells, although in different extent (Figure 6B). The strain *L. fermentum* BGSR163 exhibited higher adherence ability (13.73%), comparing to the strain *L. fermentum* BGSR227 (4.45%) (Figure 6B).

### Immunomodulatory Activity

**FIGURE 7:**
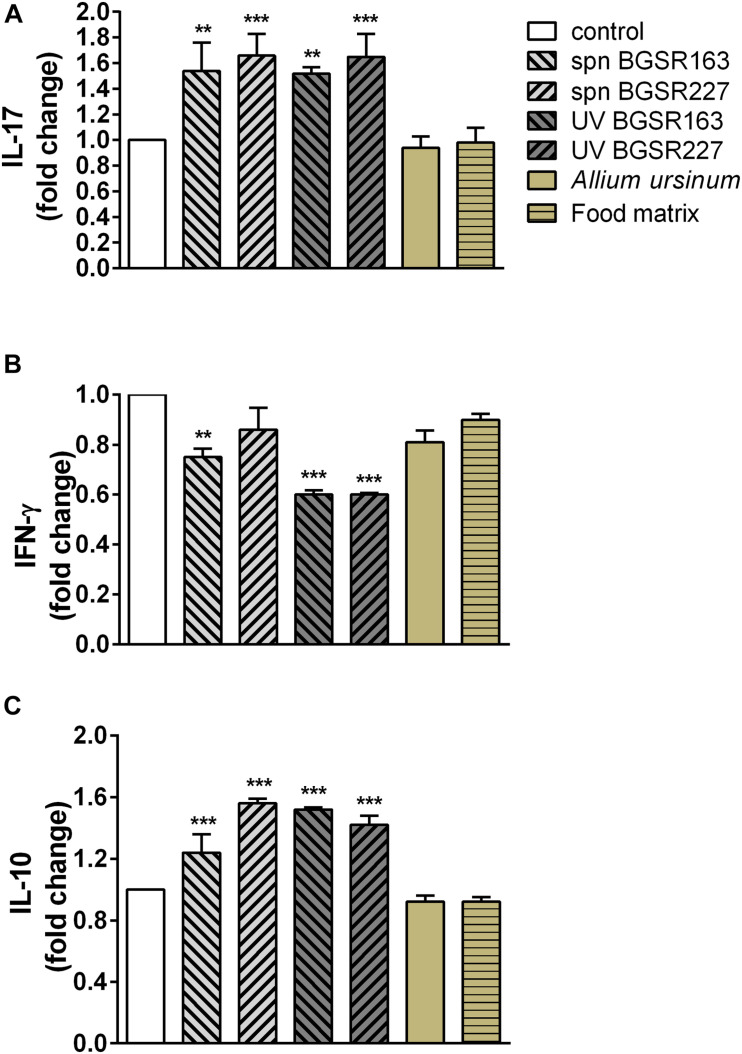
Effect of cell culture supernatant (spn) and UV inactivated *Lactobacillus. fermentum* strains BGSR163 and BGSR227, as well as *A. ursinum* and complex food matrix (commercial infant formula) after passing through the gastrointestinal tract on the production of IL-17 **(A)**, IFN-γ **(B)**, and IL-10 **(C)** on ConA stimulated MLNC after the 72 h treatment. All values are presented as mean ± SD from three independent experiments. One-way ANOVA with the Dunnett’s *post hoc* test was used to compare groups with control. The statistical significance of cytokine production is shown (**p < 0.01, ***p < 0.001).

In order to evaluate the immunomodulatory effect of potential probiotic bacteria (UV inactivated bacteria and cell culture supernatant) as well as *A. ursinum* extract, the production of proinflammatory cytokines IL-17 and IFN-γ, and immunoregulatory cytokine IL-10 of MLNC stimulated with a ConA was tested. Importantly, none of the used treatments exhibited cytotoxic effects (data not shown). Treatments of MLNC with cell-free culture supernatants and UV inactivated bacteria *L. fermentum* BGSR163 and *L. fermentum* BGSR227 significantly stimulated the production of IL-17 (p < 0.01 and 0.001 respectively) (Figure 7A), while the treatments of MLNC with cell-free culture supernatants of *L. fermentum* BGSR163 and UV inactivated bacteria of both strains led to the decreased production of IFN-γ (*p* < 0.01 and 0.001 respectively) (Figure 7B). *L. fermentum* BGSR163 and *L. fermentum* BGSR227 cell culture supernatants as well as their UV inactivated bacteria statistically significantly stimulated the production of IL-10 in MLNC (*p* < 0.001) (Figure 7C). Plant extract treatment and treatment with complex food matrix did not exert effects on cytokine production (Figures 7A–C).

## Discussion

Ramson –*Allium ursinum* has been used as a medicinal plant for centuries. While its antimicrobial, cytotoxic, and antioxidant activity were previously shown, the ramson-associated microbiota was not characterized so far. The current study represents the first attempt to elucidate the effect of simulated digestion of ramson in the presence of complex food matrix on the bioaccessibility of phenolic and organosulfur compounds as well as their *in vitro* biological activities. Hence, this paper presents the characterization of antiproliferative and antioxidant activities of ramson, together with the health-promoting properties of its associated LAB. Namely, the number of studies in the last decade identified the complex plant-associated microbial communities, so-called plant microbiota (Compant et al., 2019). Moreover, the beneficial effects of the plant microbiota on the plant growth and health was demonstrated (Compant et al., 2019). However, the comprehensive analyses of the plant extracts together with the health-promoting effects of the plant microbiota are rare. Particularly the data related to the probiotic effects of the *A. ursinum-*associated microbiota are lacking.

### Total Phenolic, Flavonoid Content, and Antioxidant Activities

Available literature data on TPC and TFC in *Allium ursinum* are very diverse and heterogeneous which is mainly caused by different procedures for extract preparation and chemical analysis. In previous study (Gitin et al., 2012) reported TPC values of 27.9 g GAE/100 g (dry basis) in leaf extract obtained by 12-day maceration with 70% ethanol, while Djurdjevic et al. (2004) determined 3.24 mg/g of total free phenolics in dry leaves extracted with 80% (v/v) boiling aqueous methanol, followed by boiling ethyl acetate. TFC content of fresh leaves was also strongly dependent on extraction method. According to Gitin et al. (2012) flavonoid content determined using ultrasound assisted extraction was 7.3 mg Quercetin/kg fresh leaves compared to 2.7 mg QE/kg using conventional maceration. In another recent study (Pavlović et al., 2017), the authors applied five different extraction techniques followed by determination of TPC and TFC in dried extracts of *A. sativum* leaves. According to obtained data TPC varied from 24.32 to 43.97 mg of GAE/g of extract and values for TFC were from 7.46 to 38.56 mg of Rutin/g of extract. Several previous studies (Nakano et al., 1981; Carotenuto et al., 1996; Wu et al., 2009) have shown that main polyphenols present in ramsons leaves are most certainly flavonoids, among them kaempferol derivatives were found to be predominant (Oszmiański et al., 2013). To the best of our knowledge not a single one of mentioned studies have not considered the bioaccessibility of ramsons polyphenol compounds. In current study total phenolic and flavonoid content have been examined in bioaccessible fraction obtained after *in vitro* digestion of fresh ramsons leaves in presence of complex food matrix. The aim of this experimental design was to mimic feeding conditions as closely as possible. Obtained results showed that the concentrations of total polyphenols and total flavonoids in bioaccessible fraction were far lower than those detected in fresh and dry leaves as well as their concentrated extracts. Determined content of TPC after the digestion in presence of food matrix was only 235–264 μg GA/mL, from which 110 μg GA/mL was present in bioacessible fraction of food matrix digested without *A. ursinum*. Similar results were obtained for TFC 40.4–55.4 μg QE/mL in samples containing *A. ursinum* and 22.7 μg QE/mL, in food matrix alone. Observed low yields were expected due to mild extraction conditions in aqueous medium of the mimicked digestive tract compared to organic solvent extraction applied in previous studies. However, it was not the only limiting factor. Various types of interactions between food constituents (proteins, carbohydrates, lipids or fibers) and polyphenols affecting their bioaccessibility have been documented previously (Stanisavljević et al., 2015; Pešić et al., 2019). When compared TPC and TFC values before (SN1t_0_–SN3t_0_) and after the digestion (SN1t_0_–SN3t_0_) a mild increase after completed digestion was observed in majority of samples containing *A. ursinum.* Similarly, in samples containing solely food matrix (SNM) increased TPC and TFC values have also been recorded upon digestion. Thus, it is not clear which portion of that increment might be attributed to release of phenolic compounds from *A. ursinum* during the digestion. Results obtained for TPC should be interpreted with the great caution especially in samples containing complex food matrices in aqueous media. It has been determined previously that Folin-Ciocalteu’s reagent is also showing reactivity toward reducing sugars, ascorbate, certain organic and fatty acids, aromatic amines and peptides which could be released during the digestion of food constituents (Prior et al., 2005). Total flavonoid content of food matrix was noticeably high, although approximately three times lower than in samples containing ramsons. This might be explained by the shortcomings of aluminum chloride based assay, which is dependent on the type of flavonoid present in the sample and are specific only for a limited number of flavonoids while it can react with phenolic acids giving non-specific absorbance at 510 nm (Pêkal and Pyrzynska, 2014). It has been shown previously that food matrix used in our study contains significant amount of phenolic acids and several flavonoids (Pešić et al., 2019). Previous studies showed that composition of food matrix and the effect of co-digestion of polyphenols may affect their antioxidant activity as well. As it can be observed from Table 4, radical scavenging activity measured by DPPH assay showed strong positive correlation with TPC (*r* = 0.93) and TFC (*r* = 0.94), while correlation of these parameters with ferrous ion chelating capacity (FCC) was far lower, *r* = 0.73 with TPC and *r* = 0.68 with TFC. DPPH scavenging activity was significantly elevated after the digestion in case of two samples SN1 an SN2 while remained unchanged for samples SN3 and SNM. Values of FCC were also increased upon completed digestion in all studied samples with the most prominent increment in food matrix digested alone (SNM). Considering these results, it can be concluded that bioaccessible constituents of *A. ursinum* did not substantially contribute to FCC, which in majority originates from food matrix and components of the digestive fluids. It can also be observed that the increase in FCC after digestion was higher in the food matrix alone than in combination with ramsons, indicating that some ramsons components interfere with the nutrient matrix components, resulting in a moderate increase in FCC. This situation has already been documented in the case of the combined digestion of polyphenols from grapes and the same type of nutrient matrix (Pešić et al., 2019). Taken altogether the quantities of TPC and TFC in bioaccessible fraction of ramson, digested in presence of food matrix were moderate, compared to one recorded previously in dry or fresh leaves and their extracts. This was expected, having in mind mild extraction conditions of simulated digestive tract and interactions of phenolic compounds with food constituents. Nevertheless, determined values of TPC and TFC were approximately two times higher in samples containing ramson than in food matrix digested alone. Results of DPPH assay were positively and strongly correlated with TPC and TFC, while the contribution of phenolic compounds to FCC was less pronounced.

**TABLE 4:** Correlations between antioxidant parameters and total phenolic and flavonoid contents.

	TPC	TFC	DPPH	FCC
TPC	1.0	0.96	0.93	0.73
TFC		1.0	0.94	0.68
DPPH			1.0	0.72
FCC				1.0

*TPC, Total phenolic content; TFC, Total flavonoid content; DPPH, 2,2-Diphenyl-1-Picrylhydrazyl; FCC, ferrous ion chelating capacity. Numbers represent *R*-square values. All shown correlations are significant at *p* < 0.05 level.*

### Qualitative Analysis of Volatile Organosulfur Compounds

According to the literature data dominant organosulfur compounds (cysteine sulfoxides) of *A. ursinum* are methiin and alliin, but it also contains certain amounts of isoalliin, propiin and ethiinas well (Sobolewska et al., 2015). Thiosulfinates are formed from cysteine sulfoxides upon tissue mechanical injury by the hydrolyticaction of the alliinase (C,S-lyase). The optimal conditions for hydrolytic cleavage of sulfoxides are pH = 6 and temperature of 35°C. The primary products of this reaction are thiosulfinates, pyruvic acid, and ammonia. The most abundant (75–90%) thiosulfinates detected in various ramson extracts were: allicin (diallylthiosulfinate = di-2-propenyl thiosulfinate) and methyl-allyl- or dimethyl thiosulfinates (Sendl and Wagner, 1991). Depending on the *Allium* species and specific conditions, thiosulfinates decomposition could yield a variety of constituents such as methyl allyl, diallyl diethyl mono-, di-, tri-, tetra-, penta-, and hexasulfides, vinyldithiins, as well as (E)- and (Z)-ajoene (Benkeblia and Lanzotti, 2007). The most abundant thiosulfinate detected previously in ramson leaf extracts are represented by: allyl methyl disulfide, diallyldisulfide, allyl methyl trisulfide, allyl (E)-1-propenyl disulfide, dimethyl trisulfide, methyl (E)-1-propenyl disulfide, allyl propyl disulfide and allyl (Z)-1-propenyl disulfide (Sobolewska et al., 2015; Pavlović et al., 2017).

Our analysis determined presence of seven volatile compounds including: 3,4-dimethylthiophene; 1,3-dithiane; diallyl disulfide; allyl methyl disulfide; 2-vinyl-1,3-dithiane; (E)-1-propenyl methyl disulfide; (E)-1-propenyl allyl disulfide. At least to the best of our knowledge, there are no available literature data on of *A. ursinum* organosulfur compounds in bioaccessible fraction obtained after *in vitro* digestion. However, similar studies have been conducted on several other *Allium* species. It has been shown previously that hydrolytic action of alliinase can be preserved in stomach especially in presence moderate to high protein meal which can transiently raise pH to 4.4 or higher, which is sufficient to enable catalysis and produce allyl-thiosulfinates (Lawson and Hunsaker, 2018). Diallyl disulfide detected in our study belongs to allyl polysulfides spontaneous transformation products of allyl-thiosulfinates (Lawson and Hunsaker, 2018). The presence of 3,4-dimethylthiophene was previously detected in *Allium sativum* essential oil from Clevenger distillation as one of major sulfur-containing compounds (Satyal et al., 2017) as well as in *A. ursinum* leaf extract as a minor compound (Ivanova et al., 2009). According to literature data 2-vinyl-1,3-dithiane is being formed during thermal degradation of aliin on temperatures above 180°C (Kubec et al., 1997) and it was also found in fried and baked garlic (Yu et al., 1994), but there is also evidence that it could be formed at 40°C (Keleş et al., 2014). This compound was recently detected in fresh leaf extracts of *A. ursinum* (Ivanova et al., 2009). Cyclic organosulfur compound reported in our study-1,3-dithiane has been characterized previously as a minor constituent of garlic with the ability to stimulate production of reactive oxygen species in neutrophils (Schepetkin et al., 2019). It was also detected in *Allium roseum*, (Zouari et al., 2012) but this is the first report in *Allium ursinum*. Allyl methyl disulfide, (E)-1-propenyl methyl disulfide, (E)-1-propenyl allyl disulfide presence was reported previously in *A. sativum* and *A. vienale* essential oil (Prabodh Satyal et al., 2017), as well in *A. ursinum* oil and leaf extracts (Schmitt et al., 2005; Gođevac et al., 2008; Sobolewska et al., 2015).

### Anticancer Properties

The results of investigation of the anticancer properties of the *Allium ursinum* extracts are in accordance with data of Xu et al. (2013) who demonstrated that watery extracts of *A. ursinum* caused apoptosis through activation of caspase-3, caspase-8 and caspase-9, and G2/M arrest in human gastric adenocarcinoma AGS cells. Our study is the first to report the influence of *in vitro* gastrointestinal digestion on the cytotoxic activity of extracts obtained from *A. ursinum* against human malignant cell lines. The extracts prepared from medicinal plant *A. ursinum* were reported to exert various pharmacological activities, such as antioxidant, antihypertensive, antidiabetic, antimicrobial, antifungal and anti-inflammatory activities in addition to anticancer activity (Xu et al., 2013; Trio et al., 2014; Sobolewska et al., 2015, and references cited therein). Use in European traditional medicine and diverse biological activities exhibited by *A. ursinum* may be attributed to various sulfur-containing compounds, including thiosulfinates which are the most abundant chemical constituents of *A. ursinum* and phenolic compounds (Xu et al., 2013; Sobolewska et al., 2015). Only seven sulfur compounds, which represent the degradation products of thiosulfinates, were detected by 2D gas chromatography analysis in the examined extracts. The allyl sulfur compounds, including diallyl disulfide identified in the tested extracts, have been shown to inhibit proliferation of malignant cells by inducing accumulation within G2/M phase as well as to induce apoptosis (Knowles and Milner, 2001; Herman-Antosiewicz and Singh, 2004; Park et al., 2007; Yi and Su, 2013). In addition, it was demonstrated that diallyl disulfide triggered apoptosis in cancer cells through activation of caspase-3 and mitochondrial signaling pathway (Nagaraj et al., 2010; Yi and Su, 2013).

Results of our *in vitro* study suggest that *A. ursinum* consumption might have health promoting properties, including anticancer effects. The extensive *in vivo* research is required to fully evaluate the possible cancer preventive efficacy of *A. ursinum* and its bioactive compounds in addition to further bioavailability and pharmacokinetic studies.

### Beneficial Effects of the *A. ursinum* Associated Microbiota

Recent literature data revealed that many of the phytotherapeutic molecules originate from plant-associated microbiota, although it is still highly unexplored area (Köberl et al., 2013). In particular, the microbiota of medicinal plants is highly specific, depending on the unique bioactive secondary metabolites produced by medicinal plants (Qi et al., 2012). Plants are acknowledged as an important source of LAB, although not systematically investigated. Recently, Köberl et al. (2019) identified the leaf surfaces and leaf endosphere of *Matricaria chamomilla* L. and *Calendula officinalis* L. as a source of LAB, with *Enterococcaceae* dominantly present in both plants, while *Lactobacillaceae* and *Carnobacteriaceae* were more abundant on *M. chamomilla*, and *Leuconostocaceae* and *Streptococcaceae* more abundant on *C. officinalis*. The specific aim of this study was the determination of *A. ursinum*-associated microbiota, as well as isolation and identification of LAB from *A. ursinum* with potential health-promoting effects.

In general, the microbial communities could be evaluated by traditional and molecular methods. The number of studies related to evaluation of ecology of microbial communities demonstrated that the diversity and structure of the associated microbiota cannot be successfully studied by using only one of the above-mentioned approaches. The conventional culture-dependent methods are time consuming and require the isolation and cultivation of microorganisms prior to their identification as well as physiological tests. Hence, the lower abundant taxa might often be outcompeted on culture plates by numerically more abundant species. In contrast, the DGGE analysis, as one of the most frequently and highly reliable molecular techniques used for the study of complex microbial ecology is based on direct DNA isolation, although this method lack quantitative information related to the proportions of the present taxa. Indeed, it is highly appreciated that the combination of both culture-dependent and culture-independent approaches should be used to describe the diversity, structure and dynamic of the complex microbial communities (Cocolin et al., 2013).

In this study the DGGE analysis revealed the predominant presence of LAB species *Lc. lactis*, *S. thermophilus*, *L. reuteri*, and *L. gasseri.* Besides, *Leuconostoc mesenteroides/pseudomesenteroides* and *Macrococcus caseolyticus*, a large Gram-positive coccus, often associated with meat and sheep, goat and cattle milk, were identified. In addition, *Erwinia billingiae*, a Gram-negative bacteria belonging to the *Enterobacteriaceae* family, usually found as a plant pathogen, as well as *Staphylococcus warneri*, associated with bacteremia, were identified in the DGGE analysis (Kamath et al., 1992; Prod’homme et al., 2017).

However, when traditional microbiological cultivation-dependent approach was used, beside LAB mainly represented by *L. fermentum*, various other species were identified including soil bacterium *Bacillus niacini* (Nagel and Andreesen, 1991), plant growth-promoting rhizobacterium *Bacillus aryabhattai* (Bhattacharyya et al., 2017), *Paenibacillus amylolyticus*, previously isolated from fermented rice bran (Akuzawa et al., 2016), plant-pathogen *Curtobacterium flaccumfaciens* (Evtushenko and Takeuchi, 2006), plant and human pathogen *Bacillus pumilus* (Yuan and Gao, 2015), opportunistic pathogen a main cause of staphylococcal infections *Staphylococcus haemolyticus* (Czekaj et al., 2015), *Arthrobacter woluwensis*, associated with subacute infective endocarditis (Bernasconi et al., 2004), as well as *Rothiaamarae* found previously in sludge of a foul water sewer (Fan, 2002).

Taken together the results obtained in this study revealed discrepancies between the two methods used, pointing that the less abundant species have not been entirely detected by culture-dependent method. The noticed discrepancies could be also related to the PCR detection of remaining DNA from dead cells that could not be detected by culture-dependant method, as well as related to the growth media used in the study, as some species have specific growth requirements that were not met in the experimental setup.

Detailed analysis of *L. fermentum* isolates revealed that all 14 strains could be divided into two PFGE pulsotypes. Hence, the health promoting potential of the strain *L. fermentum* BGSR163, belonging to pulsotype 1 and *L. fermentum* BGSR227, belonging to pulsotype 2 were further characterized.

According to (Food and Agriculture Organization of the United Nations,and World Health Organization, 2006) probiotics are defined as “live microorganisms that confer health benefits to the host when ingested in adequate amounts”. The main criteria for determination of probiotics are their safety status, survival in the simulated GIT conditions, the ability to colonize GIT and immunomodulatory potential.

The safety status of probiotic strains implies the absence of the virulence factors (e.g., hemolytic and/or gelatinase activity) and susceptibility to clinically relevant antibiotics (European Food Safety Authority, 2012). The results obtained in this study confirmed that the strains *L. fermentum* BGSR163 and *L. fermentum* BGSR227 are safe to be used as probiotics since they do not exhibit hemolytic and gelatinase activity and are susceptible to all clinically relevant antibiotics.

The survival in simulated gastrointestinal conditions is an important criterion for the selection of probiotic strains in order to exhibit their probiotic effects in the gut (Food and Agriculture Organization of the United Nations, and World Health Organization, 2006). The results of this study revealed that the strains successfully survived the simulated GIT passage. It was known from literature data that bacteria better survive the GIT transit when applied in food matrix due to the buffering and protective effect of the food components on probiotic bacteria (Ranadheera et al., 2010). Particularly, the strains successfully survived simulated mouth conditions indicating that both strains were resistant to saliva and α-amylase. The number of viable cells lowered in simulated gastric conditions, although the CFU number was still quite high, indicating that the strains are used to the acidic environment, which is expected for LAB strains (Nikolic et al., 2012). Interestingly, the growth of the strains was restored in the simulated intestinal conditions, indicating the possible pre-adaptation in the natural environment since *L. fermentum* has been commonly found in the gut.

Transitory colonization of the intestinal mucosa is another important criterion proposed by FAO/WHO guideline for selection of probiotic strains. In this study, the adhesion of *L. fermentum* strains BGSR163 and BGSR227 to Caco-2 intestinal epithelial cells (IEC) was investigated. Both strains exhibited the adherence ability to IEC in a range common for probiotic strains (Sokovic Bajic et al., 2019), although the higher adhesion was scored in strain *L. fermentum* BGSR163 than in the strain *L. fermentum* BGSR227.

Finally, the immunomodulatory effects of the strains *L. fermentum* BGSR163 and *L. fermentum* BGSR227, as well as *A. ursinum* extract, were evaluated. Both strains *L. fermentum* BGSR163 and *L. fermentum* BGSR227 significantly stimulated the production of proinflammatory cytokine IL-17 (*p* < 0.01 and 0.001 respectively), as well as the production of immunoregulatory cytokine IL-10 (*p* < 0.001). The obtained results indicate the potential of these probiotics to stimulate the immune system’s response to extracellular pathogens. Cell culture supernatant of *L. fermentum* BGSR163 and UV inactivated bacteria *L. fermentum* BGSR163 and *L. fermentum* BGSR 227 decreased the production of proinflammatory cytokine IFN-γ (*p* < 0.01 and 0.001 respectively). Given that UV inactivated strains significantly reduced the production of IFN-γ, the major Th1 cytokine, important for response to intracellular pathogens, the supernatants of cell cultures appear to better choice for use as postbiotics. Possible utilization of *L. fermentum* BGSR227 cell culture supernatant as postbiotic could be a good strategy to stimulate the response to pathogens, without reducing the ability of the immune system to respond to a concomitant viral infection. Plant extract treatment and treatment with food matrix did not exert effects on cytokine production.

## Conclusion

Taken together the results of this study suggest that *A. ursinum* consumption might have health-promoting properties, including anticancer effects. The safety status (the absence of gelatinase and hemolytic activity, susceptibility to the clinically relevant antibiotics) and health promoting potential (survival in the simulated GIT passage, the ability to colonize the intestinal mucosa and immunomodulatory potential) of *A. ursinum*-associated *L. fermentum* strains isolated in this study indicate that the strains might be used as probiotics for human consumption. However, the extensive *in vivo* research is required to fully evaluate the possible cancer preventive efficacy of *A. ursinum* and its bioactive compounds, as well as probiotic activity of its associated *L. fermentum* strains in addition to further bioavailability and pharmacokinetic studies.

## Data Availability Statement

The original contributions presented in the study are included in the article/Supplementary Material, further inquiries can be directed to the corresponding author.

## Author Contributions

NS, SB, and MT conceived and designed the study. NS and SB performed the main work. NP participated in the research of safety status and relationship between isolates’ origins, and performed experiments with the animals used. DP and AT-V participated in the isolation and enumeration of LAB. SB, MT, and DP participated in the probiotic characterization of the isolates and DGGE analysis. NS, SB, MT, and NG analyzed, interpreted, and critically revised the data. NS, SB, NG, and JS prepared the manuscript for submission. ŽJ performed antioxidant activity assays. IM tested the effects of the extracts on cell cycle phase distribution. VB performed GC × GC-MS. All authors finally approved the version to be published.

## Conflict of Interest

The authors declare that the research was conducted in the absence of any commercial or financial relationships that could be construed as a potential conflict of interest.

## Footnotes

1 http://www.ncbi.nlm.nih.gov/BLAST
