# Supplementary material for: Antioxidant and Antiproliferative Activity of Allium ursinum and Their Associated Microbiota During Simulated in vitro Digestion in the Presence of Food Matrix
Source: Front Microbiol. 2020 Dec 1;11:601616. doi: 10.3389/fmicb.2020.601616 (PMC7736176; doi:10.3389/fmicb.2020.601616)
Supplement: Supplementary file 1 [file Table_1.docx]

Supplementary Table1. Clones with the percentage of identity to known sequences in BLAST database

| **Band number** | **Strain** | [**Max Score**](https://blast.ncbi.nlm.nih.gov/Blast.cgi?CMD=Get&ALIGNMENTS=100&ALIGNMENT_VIEW=Pairwise&DATABASE_SORT=0&DESCRIPTIONS=100&DYNAMIC_FORMAT=on&FIRST_QUERY_NUM=0&FORMAT_OBJECT=Alignment&FORMAT_PAGE_TARGET=&FORMAT_TYPE=HTML&GET_SEQUENCE=yes&I_THRESH=&LINE_LENGTH=60&MASK_CHAR=2&MASK_COLOR=1&NUM_OVERVIEW=100&PAGE=MegaBlast&QUERY_INDEX=0&QUERY_NUMBER=0&RESULTS_PAGE_TARGET=&RID=KNTUD6SW015&SHOW_LINKOUT=yes&SHOW_OVERVIEW=yes&STEP_NUMBER=&OLD_VIEW=false&DISPLAY_SORT=1&HSP_SORT=1) | [**Total Score**](https://blast.ncbi.nlm.nih.gov/Blast.cgi?CMD=Get&ALIGNMENTS=100&ALIGNMENT_VIEW=Pairwise&DATABASE_SORT=0&DESCRIPTIONS=100&DYNAMIC_FORMAT=on&FIRST_QUERY_NUM=0&FORMAT_OBJECT=Alignment&FORMAT_PAGE_TARGET=&FORMAT_TYPE=HTML&GET_SEQUENCE=yes&I_THRESH=&LINE_LENGTH=60&MASK_CHAR=2&MASK_COLOR=1&NUM_OVERVIEW=100&PAGE=MegaBlast&QUERY_INDEX=0&QUERY_NUMBER=0&RESULTS_PAGE_TARGET=&RID=KNTUD6SW015&SHOW_LINKOUT=yes&SHOW_OVERVIEW=yes&STEP_NUMBER=&OLD_VIEW=false&DISPLAY_SORT=2&HSP_SORT=1) | [**Query Cover**](https://blast.ncbi.nlm.nih.gov/Blast.cgi?CMD=Get&ALIGNMENTS=100&ALIGNMENT_VIEW=Pairwise&DATABASE_SORT=0&DESCRIPTIONS=100&DYNAMIC_FORMAT=on&FIRST_QUERY_NUM=0&FORMAT_OBJECT=Alignment&FORMAT_PAGE_TARGET=&FORMAT_TYPE=HTML&GET_SEQUENCE=yes&I_THRESH=&LINE_LENGTH=60&MASK_CHAR=2&MASK_COLOR=1&NUM_OVERVIEW=100&PAGE=MegaBlast&QUERY_INDEX=0&QUERY_NUMBER=0&RESULTS_PAGE_TARGET=&RID=KNTUD6SW015&SHOW_LINKOUT=yes&SHOW_OVERVIEW=yes&STEP_NUMBER=&OLD_VIEW=false&DISPLAY_SORT=4&HSP_SORT=0) | [**E value**](https://blast.ncbi.nlm.nih.gov/Blast.cgi?CMD=Get&ALIGNMENTS=100&ALIGNMENT_VIEW=Pairwise&DATABASE_SORT=0&DESCRIPTIONS=100&DYNAMIC_FORMAT=on&FIRST_QUERY_NUM=0&FORMAT_OBJECT=Alignment&FORMAT_PAGE_TARGET=&FORMAT_TYPE=HTML&GET_SEQUENCE=yes&I_THRESH=&LINE_LENGTH=60&MASK_CHAR=2&MASK_COLOR=1&NUM_OVERVIEW=100&PAGE=MegaBlast&QUERY_INDEX=0&QUERY_NUMBER=0&RESULTS_PAGE_TARGET=&RID=KNTUD6SW015&SHOW_LINKOUT=yes&SHOW_OVERVIEW=yes&STEP_NUMBER=&WWW_BLAST_TYPE_URL=&OLD_VIEW=false&DISPLAY_SORT=0&HSP_SORT=0) | [**Per. Ident**](https://blast.ncbi.nlm.nih.gov/Blast.cgi?CMD=Get&ALIGNMENTS=100&ALIGNMENT_VIEW=Pairwise&DATABASE_SORT=0&DESCRIPTIONS=100&DYNAMIC_FORMAT=on&FIRST_QUERY_NUM=0&FORMAT_OBJECT=Alignment&FORMAT_PAGE_TARGET=&FORMAT_TYPE=HTML&GET_SEQUENCE=yes&I_THRESH=&LINE_LENGTH=60&MASK_CHAR=2&MASK_COLOR=1&NUM_OVERVIEW=100&PAGE=MegaBlast&QUERY_INDEX=0&QUERY_NUMBER=0&RESULTS_PAGE_TARGET=&RID=KNTUD6SW015&SHOW_LINKOUT=yes&SHOW_OVERVIEW=yes&STEP_NUMBER=&WWW_BLAST_TYPE_URL=&OLD_VIEW=false&DISPLAY_SORT=3&HSP_SORT=3) | **Accession** |
| --- | --- | --- | --- | --- | --- | --- | --- |
| **1** | *Macrococcus caseolyticus* strain ATCC 13548 16S ribosomal RNA gene, complete sequence | 451 | 451 | 55% | 1.00E-126 | 96% | NR_119262.1 |
| **2** | *Lactococcus lactis* subsp. *hordniae* strain NBRC 100931 16S ribosomal RNA gene, partial sequence | 702 | 702 | 78% | 0 | 99% | NR_113958.1 |
| **3** | *Lactococcus lactis* subsp. *hordniae* strain NBRC 100931 16S ribosomal RNA gene, partial sequence | 697 | 697 | 77% | 0 | 99% | NR_113958.1 |
| **4** | *Lactococcus lactis* subsp. *hordniae* strain NBRC 100931 16S ribosomal RNA gene, partial sequence | 691 | 691 | 78% | 0 | 98% | NR_113958.1 |
| **5** | *Streptococcus thermophilus* strain ATCC 19258 16S ribosomal RNA gene, complete sequence | 680 | 680 | 78% | 0 | 98% | NR_042778.1 |
| **6** | *Streptococcus thermophilus* strain ATCC 19258 16S ribosomal RNA gene, complete sequence | 680 | 680 | 78% | 0 | 98% | NR_042778.1 |
| **7** | *Streptococcus thermophilus* strain ATCC 19258 16S ribosomal RNA gene, complete sequence | 697 | 697 | 78% | 0 | 99% | NR_042778.1 |
| **8** | *Streptococcus thermophilus* strain ATCC 19258 16S ribosomal RNA gene, complete sequence | 697 | 697 | 78% | 0 | 99% | NR_042778.1 |
| **9** | *Streptococcus suis* strain ATCC 43765 16S ribosomal RNA gene, partial sequence | 545 | 545 | 77% | 6.00E-155 | 92% | NR_117504.1 |
| **10** | *Leuconostoc mesenteroides* strain ATCC 8293 16S ribosomal RNA gene, partial sequence | 713 | 713 | 79% | 0 | 99% | NR_118557.1 |
| **11** | *Lactobacillus reuteri* strain DSM 20016 16S ribosomal RNA gene, complete sequence | 682 | 682 | 78% | 0 | 98% | NR_075036.1 |
| **12** | ND |  |  |  |  |  |  |
| **13** | *Lactobacillus gasseri* strain ATCC 33323 16S ribosomal RNA gene, complete sequence | 697 | 697 | 77% | 0 | 99% | NR_075051.1 |
| **14** | *Leuconostoc pseudomesenteroides* strain LMG 11482 16S ribosomal RNA gene, partial sequence | 680 | 680 | 79% | 0 | 98% | NR_109004.1 |
| **15** | *Erwinia billingiae* strain Billing E63 16S ribosomal RNA gene, complete sequence | 688 | 688 | 76% | 0 | 99% | NR_104932.1 |
| **16** | *Staphylococcus warneri* strain AW 25 16S ribosomal RNA gene, partial sequence | 695 | 695 | 77% | 0 | 99% | NR_025922.1 |

ND – Not detected
