# Supplementary material for: Antioxidant and Antiproliferative Activity of Allium ursinum and Their Associated Microbiota During Simulated in vitro Digestion in the Presence of Food Matrix
Source: Front Microbiol. 2020 Dec 1;11:601616. doi: 10.3389/fmicb.2020.601616 (PMC7736176; doi:10.3389/fmicb.2020.601616)
Supplement: Supplementary file 2 [file Table_2.DOCX]

Supplementary Table 2. Bacterial isolates identified by 16S rDNA sequencing; G - gelatinase positive strains, C – catalase positive strains

| Medium | Strain |  |  | 16S seq |
| --- | --- | --- | --- | --- |
| Nutri | BGSR7 | G | C | *Bacillus niacini* |
|  | BGSR18 |  | C | *Curtobacteriumflaccumfaciens* |
|  | BGSR22 |  | C | *Staphylococcus haemolyticus* |
|  | BGSR23 |  | C | *Curtobacterium sp.* |
|  | BGSR25 | G | C | *Curtobacteriumflaccumfaciens* |
|  | BGSR59 | G | C | *Bacillus aryabhattai* |
|  | BGSR63 | G | C | *Bacillus aryabhattai* |
|  | BGSR87 |  | C | *Curtobacteriumflaccumfaciens* |
|  | BGSR89 |  | C | *Staphylococcus haemolyticus* |
|  | BGSR90 |  | C | *Staphylococcus warneri* |
|  | BGSR179 |  | C | *Staphylococcus warneri* |
| GM17 | BGSR29 | G | C | *Paenibacillusamylolyticus* |
|  | BGSR32 | G | C | *Bacillus pumilus* |
|  | BGSR34 | G | C | *Arthrobacterwoluwensis* |
|  | BGSR35 | G | C | *Paenibacillus borealis* |
|  | BGSR37 | G | C | *Curtobacteriumflaccumfaciens* |
|  | BGSR38 |  | C | *Staphylococcus haemolyticus* |
|  | BGSR48 |  | C | *Paenibacillus sp.* |
|  | BGSR52 |  | C | *Staphylococcus pasteuri* |
|  | BGSR100 | G | C | *Rothiaamarae* |
|  | BGSR105 | G | C | *Arthrobacterwoluwensis* |
|  | BGSR140 | G | C | *Bacillus circulans* |
|  | BGSR141 | G | C | *Bacillus thuringiensis/cereus/subtilis* |
|  | BGSR147 |  | C | *Staphylococcus haemolyticus* |
|  | BGSR201 |  | C | *Staphylococcus epidermidis* |
|  | BGSR204 | G | C | *Staphylococcus epidermidis* |
|  | BGSR212 | G | C | *Bacillus paralicheniformis* |
| MRS | BGSR148 |  |  | *Lactobacillus fermentum* |
|  | BGSR163 |  |  | *Lactobacillus fermentum* |
|  | BGSR223 |  |  | *Lactobacillus fermentum* |
|  | BGSR224 |  |  | *Lactobacillus fermentum* |
|  | BGSR225 |  |  | *Lactobacillus fermentum* |
|  | BGR226 |  |  | *Lactobacillus fermentum* |
|  | BGSR227 |  |  | *Lactobacillus fermentum* |
|  | BGSR229 |  |  | *Lactobacillus fermentum* |
|  | BGSR230 |  |  | *Lactobacillus fermentum* |
|  | BGSR231 |  |  | *Lactobacillus fermentum* |
|  | BGSR232 |  |  | *Lactobacillus fermentum* |
|  | BGSR236 |  |  | *Lactobacillus fermentum* |
|  | BGSR238 |  |  | *Lactobacillus fermentum* |
|  | BGSR239 |  |  | *Lactobacillus fermentum* |
|  | BGSR242 |  |  | *Lactobacillus fermentum* |
|  | BGSR243 |  | C | *Staphylococcus warneri/pasteuri* |
|  | BGSR245 |  | C | *Staphylococcus pasteuri* |
|  | BGSR250 |  | C | *Staphylococcus warneri* |
|  | BGSR253 |  | C | *Staphylococcus warneri/pasteuri* |
|  | BGSR254 |  | C | *Staphylococcus warneri/pasteuri* |
|  | BGSR262 |  | C | *Staphylococcus warneri* |
|  | BGSR265 |  | C | *Staphylococcus warneri* |
|  | BGSR266 |  | C | *Staphylococcus warneri/pasteuri* |
